# Central Sensitization Syndrome and the Initial Evaluation of a Patient with Fibromyalgia: A Review

**DOI:** 10.5041/RMMJ.10204

**Published:** 2015-04-29

**Authors:** Kevin C. Fleming, Mary M. Volcheck

**Affiliations:** 1 Assistant Professor of Medicine, College of Medicine; Division of General Internal Medicine, Section of Complementary and Integrative Medicine, and Fibromyalgia and Chronic Fatigue Clinic, Mayo Clinic, Rochester, Minnesota, USA;; 2 Nursing in Fibromyalgia/Pain Rehabilitation Center, Mayo Clinic, Rochester, Minnesota, USA

**Keywords:** Central sensitization, fibromyalgia, medically unexplained symptoms

## Abstract

In both primary care and consultative practices, patients presenting with fibromyalgia (FM) often have other medically unexplained somatic symptoms and are ultimately diagnosed as having central sensitization (CS). Central sensitization encompasses many disorders where the central nervous system amplifies sensory input across many organ systems and results in myriad symptoms. A pragmatic approach to evaluate FM and related symptoms, including a focused review of medical records, interviewing techniques, and observations, is offered here, giving valuable tools for identifying and addressing the most relevant symptoms. At the time of the clinical evaluation, early consideration of CS may improve the efficiency of the visit, reduce excessive testing, and help in discerning between typical and atypical cases so as to avoid an inaccurate diagnosis. Discussion of pain and neurophysiology and sensitization often proves helpful.

## INTRODUCTION

Physicians often see patients who have chronic widespread pain. Many also have multiple non-specific symptoms that lack a firm diagnosis. Fibromyalgia and medically unexplained symptoms (MUS) result in considerable psychosocial impairment, work disability, and increased health care utilization by patients.^1^–^3^ For example, the annual health care costs incurred by those with chronic pain, excluding costs for surgical procedures, average $12,900 to $18,833.^4^ For patients with mild, moderate, and severe fibromyalgia, annual direct health care resource costs were estimated at $4,854, $5,662, and $9,318 per patient, respectively. Annual mean indirect costs (including absenteeism, unemployment, early retirement, and disability) for subjects with mild, moderate, and severe fibromyalgia were estimated at $4,428, $14,664, and $29,996, respectively.^5^ In spite of these expenditures, and lacking successful treatment, patients are often dissatisfied.^6^ Furthermore, the challenges of fibromyalgia (FM) can leave practitioners feeling overwhelmed to the point of burnout.^6^,^7^ This article addresses the initial patient evaluation in the setting of FM and provides guidelines for interviewing techniques and observations. In addition, the connection between FM and the sensory amplification disorder known as central sensitization syndrome is explored.^8^

An illustrative patient may be a 43-year-old woman with a 7-year history of chronic diffuse myalgias and arthralgias, foggy thinking, and fatigue. She has been recently seen in the emergency department for a 2-month history of continuous chest pressure and a sense of being unable to take a full breath. In that setting, cardiac and pulmonary testing excluded myocardial infarction, stable or unstable angina, and any evidence of pulmonary disease. Prior outpatient evaluations for the chronic symptoms have been inconclusive. She is seeking a second opinion.

## CENTRAL SENSITIZATION

There is considerable overlap among syndromes such as fibromyalgia, chronic fatigue, irritable bowel syndrome, chronic pelvic pain, and chronic daily headache. Many patients carry multiple diagnoses simultaneously (Box 1).^9^,^10^ Myalgias, fatigue, sleep disorder, and depression are present both in FM and chronic fatigue.^11^ Similarly, patients with chronic fatigue syndrome, FM, and temporomandibular disorder share key symptoms and frequently report concurrent irritable bowel syndrome, multiple chemical sensitivities, atypical facial pain, and tension headache.^12^ There is substantial overlap among the case definitions and diagnostic criteria for these disorders, even for core symptoms. In a review of functional somatic syndromes, the criteria for multiple disorders contained “bloating,” “headache,” “fatigue,” and “abdominal pain.”^11^,^13^ Indeed, the principal diagnosis largely depends on the patient’s cardinal complaint, most worrisome symptom, or the clinician’s subspecialty.^13^ Although several terms have been used to discuss the clustering of these conditions, this phenomenon is increasingly understood as a manifestation of the underlying syndrome of central sensitization.

Box 1. **Diagnoses, Self-diagnoses, and Symptoms that May Suggest Central Sensitization Syndrome (Especially If Copious)**

Abdominal bloating
Abdominal pain, chronic abdominal pain
Adrenal insufficiency (self-diagnosed), adrenal fatigue
Alopecia, hair loss, trichotillomania
Anxiety
Atypical facial pain
Atypical or non-cardiac chest pain
Autoimmune disorder (self-diagnosed)
Autonomic disorder (self-diagnosed)
Black mold, toxic black mold (self-diagnosed)
Brain fog, fibrofog
Burning mouth syndrome
Burning tongue
Candida or chronic yeast infection
Chiari malformation
Chronic low-back pain
Chronic non-specific lightheadedness
Chronic pain
Chronic pelvic pain
Chronic prostatitis
Chronic tension or migraine headaches
Chronic testicular or scrotal pain
Chronic whiplash-associated disorders
Chronic widespread pain
Complex regional pain syndrome
Delusions of parasitosis
Depression or bipolar disorder
Dizziness
Edema or swelling complaints not evident on examination
Ehlers–Danlos syndrome
Fatigue or chronic fatigue
Fibromyalgia, myalgic encephalitis
Hormone imbalance
Hyperventilation
Hypoglycemia (self-diagnosed)
Immune deficiency (self-diagnosed)
Interstitial cystitis, painful bladder syndrome
Irritable bowel syndrome
Joint pains
Low testosterone or hypogonadism (with normal test results)
Lupus (self-diagnosed)
Lyme disease, chronic Lyme disease (self-diagnosed)
Meniere disease
Morgellons disease (self-diagnosed)
Multiple chemical sensitivities
Multiple drug allergies or intolerances (self-diagnosed)
Multiple food allergies or intolerances (self-diagnosed)
Myofascial pain syndrome
Palpitations
Panic disorder, episodes, attacks
Pelvic pain, chronic pelvic pain, premenstrual syndrome
Polycystic ovary syndrome
Porphyria (self-diagnosed)
Post-deployment syndrome
Post-traumatic stress disorder
Postural orthostatic tachycardia syndrome (POTS)
Pseudotumor cerebri
Schamberg disease, soft tissue tumors
Sick building syndrome
Sjögren syndrome (blamed for multiple symptoms)
Temporomandibular disorders, temporomandibular joint pain
Thyroid disease (with normal test results, usually self-diagnosed)
Tinnitus
Vulvodynia, vulvar vestibulitis

Central sensitization can be defined as a state in which the central nervous system amplifies sensory input across many organ systems. This enhanced response to sensation includes plasticity at a neuronal level that increases sensitivity for future stimulation. Heightened sensitivity results in the perception of pain from non-painful stimuli (allodynia) and greater pain than would be expected from painful stimuli (hyperalgesia). Prominent visceral hypersensitivity can affect every organ system and produce intolerable discomfort. Ultimately, all of these amplified sensations, which range from the arthralgias and myalgias of FM, to headaches, abdominal complaints, and pelvic concerns, may constitute an initial presentation of CS as MUS.^8^,^14^

On a cellular level, CS results from multiple processes altering the functional status of nociceptive neurons. These processes include increases in membrane excitability, facilitation of synaptic strength, and decreases in inhibitory transmission (disinhibition). Affected neurons display spontaneous activity, reduced activation threshold, and enlarged receptive fields. Hypersensitivity amplifies the sensory response elicited by normal inputs such as innocuous stimuli and normal body sensations. Perceptions may no longer be coupled to the intensity, duration, or even the presence of noxious peripheral stimuli.^15^ And yet, CS results in changes in brain activity that can be detected by functional magnetic resonance or positron emission tomographic imaging and electro-physiologic studies.^8^ In this paper, we will focus on FM, which is an archetypal presentation of CS.^16^

## SPECIFIC INTERVIEWING TECHNIQUES AND OBSERVATIONS

Following introductions, the duration available for the visit can be made explicit. If the patient has external medical records, the physician can acknowledge that because of limited time these may need complete review afterward. Although interviewing the patient alone is preferable, sometimes the patient wishes to be accompanied by spouse, family member, or friend. This may interfere with full engagement between the clinician and the patient if the companions are numerous or tend to interrupt the dialogue. However, if the patient insists on being accompanied, then the clinician should acquiesce while limiting the number of additional persons. If interruptions or other difficulties ensue, it can be useful to say: “It is always best for me to hear first from my patient. Let’s try that route first, and if we need your help in clarifying some things, then that can come later.” This maintains the focus on the patient’s concerns and the evolving relationship with the new clinician.

Patients who have undergone multiple medical evaluations have often developed a highly medicalized personal story, dominated by a reporting of procedures and test results. Some have even settled on a diagnosis and want the clinician to perform a desired test, or treat it with a specific intervention. Traditional open-ended questions in this setting may be resisted as the patient prefers a shortcut to the test or treatment as opposed to further dialogue. Other patients may present equivalent challenges to the usual open-ended question technique by virtue of cultural differences, educational levels, maladaptive personality styles, or co-morbid illnesses. Given the usual time constraints, however, more directive interviewing must occur so as to determine quickly the most salient historical data.

In this regard, understanding the timing of symptom onset is crucial. Rather than asking simply about duration, it can be useful to inquire more specifically: “When did you first start having pain? Grade school, high school, or later?” These questions may prompt the patient to recall forgotten episodes from early childhood, or in the wake of a past illness (e.g. infectious mononucleosis), or important stressors (e.g. sexual assault, car accident, death of a family member).

Particular attention should be given to the reporting of symptoms that may be correlates of unpleasant and unacknowledged emotional states. These symptoms might include neuropsychiatric problems such as sleep disturbance, decreased concentration, poor memory, “mental fog,” tremors, and lightheadedness.^17^ The patient’s history of drug trials, chronic narcotic use, injections, surgical or other procedural interventions, and non-traditional approaches merits exploration to ascertain whether benefit was achieved. Chronic narcotic use, in particular, is noteworthy as an inducer of unremitting hyperalgesia.^18^ Lastly, the presence of disability, unemployment, drug or interpersonal abuse, or a family history of mental illness can usually be obtained through a standard patient questionnaire.

## PAIN BEHAVIOR

During the interview a patient with FM and other chronic pain disorders may exhibit notable pain behaviors which have been described as communicative or protective (Box 2). Communicative pain behaviors include non-verbal facial expressions such as grimacing, wincing, or crying, as well as verbal or paraverbal pain expressions such as words, grunts, sighs, and moans. Protective pain behaviors include touch avoidance, anticipatory flinches, and movements such as guarding or holding the painful area. “Protective” also entails therapeutic maneuvers, such as moving or rubbing the painful area of the body, rocking, weight shifting, or repeatedly standing up, arching the back, walking around, or even lying on the exam floor (Box 3).^19^

Box 2. **Pain Behaviors**

Grimacing, wincing
Crying
Holding or rubbing the affected area
Rocking
Fidgeting, shifting weight in a chair
Repeatedly standing and/or walking
Arching the back or neck
Anticipatory flinch or withdrawal
Need to lie down

Box 3. **Non-verbal and Verbal Communication: Possible Red Flags**

Eyes closed during the interview
Lights turned off
Patient lying down when the physician enters the room
Clothing (sunglasses,^20^ hoodie, baseball cap, age-inappropriate)
Stuffed animals (brought and carried by an adult patient)
Angry or irritable
Crying
A forced or tight smile
Eye rolling
Sighs when asked questions

## EMOTIONAL BEHAVIOR

Patients with FM and other chronic pain disorders may exhibit emotional behavior ranging from apathy to anger. Neutral, virtually expressionless postures may be maintained while discussing issues of pain, stress, loss, or trauma. Some patients may speak of their severe symptoms and disabilities with such inappropriate nonchalance as to suggest *la belle indifference.*^21^ Others may act with anger or hostility from the outset with observed behaviors including: curt responses, frowns, scowls, cynical remarks, profanities, eye rolling, and direct criticism of the skills of past and present providers, including the interviewer.

It is essential to maintain equanimity in the face of both apathetic and angry provocations, and to exhibit empathy and forbearance rather than defensiveness, confrontation, or rejection. Confronting the frustration of a patient’s apathy or anger with the same usually exacts an enormous cost in time and emotional effort. If the patient cannot be effectively engaged or redirected, the clinician must avoid responding defensively, focus on completing the interview, and possibly terminate the session if behaviors prevent further dialogue.^22^

## CO-MORBID PSYCHIATRIC DISORDERS AND TRAUMA

Individuals presenting with fibromyalgia often demonstrate high levels of self-critical perfectionistic behavior. This chronic form of psychosocial stress includes an internalized sense of helplessness or hopelessness and ultimately increases fatigue, depression, and pain awareness while diminishing health and longevity.^23^–^25^ Fibromyalgia patients frequently suffer anxiety disorders, sleep disorders, and personality disorders as well.^9^,^26^–^29^

Additionally, there is a tendency for FM patients to share histories of early life physical or sexual abuse;^30^ of assault, neglect, alcoholic parents, and physical trauma;^31^,^32^ and of various catastrophic events such as war, torture, floods, and other causes of post-traumatic stress disorder.^33^–^35^ Indeed, several studies have reported that patients who have a history of adverse childhood experiences or post-traumatic stress disorder or are victims of intimate partner violence often have multiple somatic complaints and an increased prevalence of both functional and chronic illnesses.^36^–^44^

Although the connection between trauma and FM or somatic symptoms is an important one, the clinician must be judicious when approaching this topic. Many patients have already undergone considerable psychological work in efforts to address these issues and quite reasonably balk at the idea of resurrecting these memories and feelings with a newly encountered clinician. Even if no such treatment has yet been undertaken, an initial visit is usually not the time to search fully for this possibility. Instead, it is better to revisit this arena during a later appointment, or to defer it to other clinicians further on in the evaluation process when the patient will more likely feel safer and assured of the team’s good intention. In the first visit, it is sufficient for a physician to be aware that the story behind any patient with FM is almost always “fraught with background.”^45^

Notably, FM patients with multiple unexplained symptoms must be evaluated without an expectation of attributing their difficulties to mental illness. The recently updated Diagnostic and Statistical Manual of Mental Disorders (DSM-V) has replaced the previous edition’s (DSM-IV) *somatoform disorders* grouping with the current *somatic symptom and related disorders* section.^46^ The stated intent of this change was to avoid a mental disorder diagnosis only on the basis of undiagnosed somatic symptoms. Instead, an emphasis upon abnormal patient responses to positive symptoms and signs, whether explained or not, is their critical feature.^1^,^46^

## MENTAL FOG

Patients with FM often complain of cognitive difficulties. This may even be observed in the initial interview. These states are characterized as sensations of being in a daze or mental fog, sometimes referred to as “fibrofog.” Patients may report forgetting conversations, phone numbers, plans, and activities. They may note feeling lost in familiar places, being unable to carry out simple tasks like grocery shopping, or finding complex tasks like driving almost impossible.^47^ Formal cognitive testing in these patients is often within normal limits overall but also may reveal patchy attention deficits. It is a situation in which impaired mental function appears mostly to come from a compromised capacity for focusing attention, for processing and remembering new sensory data, and for then performing complex tasks. This patchy attention focus impairs memory formation since new data are not collected with clarity or stored reliably.^48^ Clinician awareness and recognition of this phenomenon can further support consideration of CS during initial contacts with FM patients.

## DRUG AND FOOD INTOLERANCE

Patients with FM and somatic symptoms frequently note many medications to which they are allergic or intolerant. This practice has been termed *multiple drug intolerance syndrome* and is characterized by a listing of non-allergic hypersensitivity reactions to chemically unrelated agents. The reactions are not associated with abnormalities on skin prick and patch tests or with measurement of specific increased IgE levels. Additionally, the same patients may complain of multiple food allergies, sensitivities, or intolerances.^49^ Many have adopted special diets, such as gluten-free, vegan, or lactoseavoidant regimens, in an attempt to reduce their symptoms. In the most severe cases, malnutrition and considerable weight loss have resulted. Similar multisystem symptoms of intolerance or hypersensitivity to specific environmental exposures occur in individuals reporting multiple chemical sensitivity, noise sensitivity, sick building syndrome, and general environmental intolerance. Multiple drug, food, and environmental intolerances are strongly suggestive of a CS role.^50^,^51^

## APPROACH TO ACCOMPANYING SYMPTOMS

The number, duration, severity, and often disabling impact of somatic symptoms in FM patients may cause considerable worry for the clinician who hopes to avoid missed diagnoses and unnecessary testing. It is impossible to investigate fully every symptom or complaint. Clearly, another approach is needed.

One useful paradigm from statistical analysis is that of common-cause variation versus special-cause variation. The former is the background noise inherent in a given process and described as usual or random. The latter is not inherent in a given process but rather is unusual and non-random with an often-assigned specific cause.^52^ The distinction between common-cause and special-cause variation is useful when considering whether the patient with MUS is typical or atypical.

With sufficient experience and a recognition of the shared features among MUS patients with CS conditions, most clinicians realize soon during the initial visit that they are likely to diagnose the patient with some variant of CS. The typical combinations of oversized record packets, pain behaviors, conjoined apathy and anger, trauma histories, mental fog, psychiatric co-morbidities, and food or drug intolerances provide a substrate upon which the clinician can confidently consider whether an individual patient’s variation from others is more likely “common” and random or “special” and non-random—that is, typical or atypical.

Symptoms that are judged to be atypical of CS can be considered as special-cause variations and merit further investigation. For example, abnormal weight loss, drenching night sweats, observed syncope or seizures, nocturnal or bloody diarrhea, and radiculopathic dysesthesias or weakness imply non-random specific causes, even in an otherwise typical CS context. On the other hand, in the context of fairly typical symptoms for CS, then only limited testing need be considered (Table 1).

**Table 1.** Assessments to Consider in Patients with Fibromyalgia or Medically Unexplained Symptoms.

**Assessment Tool or Test**		**Indication**
**Questionnaires**	Generalized Anxiety Disorder 7-item questionnaire	Anxiety
	Patient Health Questionnaire 9-item assessment	Depression
**Laboratory Studies**	Complete blood cell count	Fatigue
	Aspartame aminotransferase and alanine aminotransferase	Fatigue
	Bilirubin	Pruritus
	Hemoglobin A_1c_ and fasting glucose	Fatigue, burning mouth
	Vitamin B_1_, B_2_, B_6_, B_12_	Paresthesias, burning mouth
	Vitamin C, ascorbic acid	Burning mouth
	Vitamin D	Pain
	Folate	Burning mouth
	Ferritin	Burning mouth, pruritus
	Thyroid-stimulating hormone	Fatigue, burning mouth
	Testosterone (men)	Fatigue
	Morning cortisol	Fatigue
	Sedimentation rate, C-reactive protein	Arthralgias, myalgias
	Complement, total and C4	Pruritus
	Antinuclear antibody	Burning mouth
	Sjögren syndrome antigen A and B	Burning mouth
	Immunoglobulins A, M, G	Burning mouth, possible immunodysfunction
	Tissue transglutaminase antibodies	Food intolerance, irritable bowel, diarrhea
	Protein electrophoresis	Paresthesias, pruritus
	Heavy metals	Paresthesias
	Metanephrines, serum	Indeterminate spells
**Tests**	Echocardiogram	Exertional fatigue
	Electromyogram	Paresthesias
	Holter monitor	Palpitations, non-specific dizziness, lightheadedness
	Overnight oximetry	Fatigue
	Spirometry with bronchodilator	Dyspnea
	Tilt table test	Postural orthostatic tachycardia syndrome, chronic fatigue, lightheadedness

Laboratory evaluation of FM should include a complete blood count, liver transaminases, fasting glucose/hemoglobin A1c, creatinine, thyroid stimulating hormone, vitamin D, erythrocyte sedimentation rate, and C-reactive protein. Unless symptoms or signs (e.g. swollen joints) suggest a rheumatologic disorder, the antinuclear antibody, cyclic citrullinated peptide, and rheumatoid factor assays are generally not recommended. Barring pain symptoms atypical for fibromyalgia, radiologic imaging is also generally unnecessary.^16^ In our Fibromyalgia Clinic, we also have the patient complete several questionnaires to assess anxiety, depression, sleep, the performance of daily activities, and the severity, location, and duration of pain (Table 2).

**Table 2.** Useful Fibromyalgia Questionnaires.

**Questionnaires**
Screening for Generalized Anxiety Disorder (GAD)
Widespread Pain Index (WPI; for the 2010
Fibromyalgia Diagnostic Criteria)
Symptom Severity score (SS score; for the 2010
Fibromyalgia Diagnostic Criteria)
Berlin Questionnaire (for sleep apnea)
Activities of Daily Living screen (level of impairment in performing ADLs: None, mild, moderate, severe), or the Sheehan Disability Scale (SDS)

## CLOSING AND SUMMARIZING

At the close of the initial visit, the physician should deliver a concise summary statement that includes a synopsis of the history, past investigations and treatment, and present plans for next steps. The clinician can point to the many negative results from previous evaluations while making sure that common diagnoses have not been missed. If the overall assessment remains consistent with a typical FM presentation, then a discussion of CS can be initiated.

Indeed, explaining how the patient can be experiencing chronic pain and other symptoms in the absence of anatomical causes or abnormal tests is key to gaining acceptance of the diagnosis. In our practice, an electronic slideshow is used by the physician to guide the patient through the discussion of pain and central sensitization, tailored to the patient’s educational level, specific symptoms, and life experiences (Table 3). This can take from 15 to 20 minutes, and could instead be performed by a nurse educator, or in a recorded format if preferred. Understanding the pain processing disorder of CS provides a useful cognitive anchor for the patient, and helps allay the common concern that their symptoms are being dismissed as “all in their head” (i.e. imaginary), malingering, or reflecting a mental illness.^53^ In addition, educating FM patients about pain physiology can also improve endogenous pain inhibition and pain-free movement performance.^54^ Pain physiology education can modify maladaptive approaches to pain, limit catastrophic thinking, and reduce pain behaviors.^55^ If fibromyalgia is confirmed, patients in our clinic are referred to a 1.5-day treatment program that was developed at our institution, based on non-pharmacologic treatment principles for chronic pain from our intensive three-week Pain Rehabilitation Program. It is coordinated with evaluation and treatment protocol sessions. The 1.5-day program targets the cognitive aspect of fibromyalgia, rather than focusing on pain. It addresses negative thinking, maladaptive emotions and cognitions, avoidance behaviors, and catastrophizing. Hypervigilance, health anxiety, and somatization are reviewed. Cognitive retraining techniques, meditation, physical activity approaches, medications, narcotics, social skills, returning to work, and other topics are discussed (Table 4).

**Table 3.** Education Session: the Neurophysiology of Pain.

**How Acute Pain Originates in the Body**
Peripheral nerves, ascending pain pathways
The role of the thalamus, descending pain pathways
The somatosensory region
Pain processing (physical, emotional) and pain memory
The limbic system: (i) the fight or flight response; (ii) threat surveillance
How threat modifies sensation
The hypothalamic-pituitary-adrenal (HPA) axis
Chronic activation of the limbic system
Pain and sensory amplification/sensitization
Brain imaging of modified pain processing in fibromyalgia (FM)
Brain imaging of modified pain networks in FM
Reduced endorphin receptors in fibromyalgia
Brain imaging comparing normal injury pain to FM
Hyperalgesia and allodynia
Recruitment
Neurochemical changes in central sensitization (CS)
Changes in the HPA axis in CS
Peripheral sensitization
Narcotic effects on pain in FM/CS
CS autonomic effects
CS motor effects
Chronic fatigue brain changes
Frontal brain and limbic system control
Neuroplasticity and recovery

CS, central sensitization; FM, fibromyalgia; HPA, hypothalamic-pituitary-adrenal.

**Table 4.** Twelve-Hour Fibromyalgia Treatment Program.^58^

**Education (Completed by Registered Nurse, with Wellness Specialist for the Exercise Component)**
**Diagnosis and possible contributing factors**
Epidemiology
Signs and symptoms
Diagnosis
Proposed models of pathophysiology
**Self-management**
Definition of self-management, focusing on what you can control
Discuss the downward “cycle of pain”
Learn new coping techniques to break out of the downward spiral
Cognitive behavioral techniques, including confronting negative or perfectionistic tendencies and replacing them with more realistic and positive tendencies
Education on stress management, relaxation, sleep hygiene, communication, energy conservation, time management, step-graded exercise, nutrition, mental fog, forgiveness, grief, humor, and planning of a difficult day
Identifying patterns of “pushing through the pain” followed by a “crash and burn” tendency
Modify behavior utilizing time management and planning daily activities
**Practice sessions:** Breakout sessions provided to practice specific relaxation techniques with the registered nurse, practice stretching and exercise education with the wellness specialist, and goal setting session with the registered nurse to set a plan in place for returning home

In a 12 month follow-up study of patients experiencing this brief treatment program, there was significant improvement in symptoms, quality of life, and ability to function compared with pretreatment measures. Notably, there was no significant decline between six and 12 month reassessments, except for depression. This finding was consistent with other fibromyalgia studies which also demonstrated positive health outcomes. Patient characteristics associated with a greater response to treatment were: younger age, college or higher level of education, higher baseline depression score, fewer tender points on physical examination, and the lack of abuse history. However, duration of symptoms, gender, opioid use, smoking, marital status, and employment status did not affect treatment response.^56^,^57^

These core self-management strategies are a critical component in fibromyalgia treatment. Although certain medications may provide relief for some symptoms, these are more likely to be successful as an adjunct to an integrated approach with non-pharmacological therapies including self-management, exercise therapy, and cognitive behavioral therapy (Table 5). Serotonin norepinephrine reuptake inhibitors (duloxetine, milnacipran), gabapentinoids (pregabalin, gabapentin), and γ-hydroxybutyrate have demonstrated efficacy in the treatment of fibromyalgia.^16^ The tricyclic agents (nortriptyline, amitriptyline) have also been shown to be effective, but recent concerns about the long-term use of medications with anticholinergic effects and incident dementia have been raised.^58^ Opioids are best avoided in treating chronic FM pain because they work well in only a third of patients and often worsen pain over time (opioid-induced hyperalgesia).^59^

**Table 5.** Non-pharmacologic Treatments for Fibromyalgia.

**Treatments**
Patient education
Cognitive behavioral therapy
Biofeedback
Mind–body techniques
Meditative movement therapies (tai chi, yoga, qigong)
Paced breathing/meditation
Complementary therapies (myofascial release massage, acupuncture)
Creative work (art, music, dance therapy)
Workbooks (anxiety, post-traumatic stress disorder, behavior modification)
Graded aerobic exercise
Water-based exercise
Strength training
Hypnotherapy
Chiropractic manipulation
Transcutaneous electrical nerve stimulation
Sleep hygiene

## CONCLUSION

The initial evaluation of the patient with FM, especially when complicated by other somatic symptoms, can be stressful. Many of these patients are ultimately diagnosed with conditions associated with CS. Central sensitization is a state in which the central nervous system amplifies sensory input across many organ systems and causes a myriad of symptoms. A focused review of medical records and specific interviewing techniques and observations are critical tools for identifying the most important symptoms. An ongoing consideration of CS may improve the efficiency of the visit, reduce excessive testing, and help in discerning between typical and atypical MUS cases to avoid an inaccurate diagnosis. A discussion of symptom neurophysiology often proves quite helpful in patient management.

## Abbreviations:

CS: central sensitization;
FM: fibromyalgia;
MUS: medically unexplained symptoms.
